# A Qualitative Study of Self and Caregiver Perspectives on How Autistic Individuals Cope With Trauma

**DOI:** 10.3389/fpsyt.2022.825008

**Published:** 2022-07-14

**Authors:** Elise Ng-Cordell, Anika Rai, Hira Peracha, Tamara Garfield, Stephen E. Lankenau, Diana L. Robins, Steven J. Berkowitz, Craig Newschaffer, Connor M. Kerns

**Affiliations:** ^1^Department of Psychology, University of British Columbia, Vancouver, BC, Canada; ^2^A. J. Drexel Autism Institute, Drexel University, Philadelphia, PA, United States; ^3^Department of Community Health and Prevention, School of Public Health, Drexel University, Philadelphia, PA, United States; ^4^School of Medicine, University of Colorado, Aurora, CO, United States

**Keywords:** trauma, autism (ASD), coping (C), quantitative analysis, internalizing

## Abstract

**Background:**

Coping can moderate the relationship between trauma exposure and trauma symptoms. There are many conceptualisations of coping in the general population, but limited research has considered how autistic individuals cope, despite their above-average rates of traumatic exposure.

**Objectives:**

To describe the range of coping strategies autistic individuals use following traumatic events.

**Methods:**

Fourteen autistic adults and 15 caregivers of autistic individuals, recruited via stratified purposive sampling, completed semi-structured interviews. Participants were asked to describe how they/their child attempted to cope with events they perceived as traumatic. Using an existing theoretical framework and reflexive thematic analysis, coping strategies were identified, described, and organized into themes.

**Results:**

Coping strategies used by autistic individuals could be organized into 3 main themes: (1) Engaging with Trauma, (2) Disengaging from Trauma, and (3) Self-Regulatory Coping. After the three main themes were developed, a fourth integrative theme, Diagnostic Overshadowing, was created to capture participants' reports of the overlap or confusion between coping and autism-related behaviors.

**Conclusions:**

Autistic individuals use many strategies to cope with trauma, many of which are traditionally recognized as coping, but some of which may be less easily recognized given their overlap with autism-related behaviors. Findings highlight considerations for conceptualizing coping in autism, including factors influencing how individuals cope with trauma, and how aspects of autism may shape or overlap with coping behavior. Research building on these findings may inform a more nuanced understanding of how autistic people respond to adversity, and how to support coping strategies that promote recovery from trauma.

## Introduction

Psychological trauma can be defined as events or circumstances that are experienced by an individual as physically or emotionally harmful or life-threatening, and that are associated with lasting adverse effects on functioning and well-being substance abuse and mental health services administration (SAMHSA) (1). This broad definition underscores that what is traumatic varies from one person to another. Moreover, trauma results not only from an event or circumstance itself, but also from the way one appraises and copes with that event or circumstance. Traumatic reactions are similarly variable, encompassing a wide range of emotional and behavioral outcomes including but not limited to post-traumatic stress disorder (PTSD) – a specific set of stress, intrusion, avoidance, mood, or arousal symptoms that persist for over a month after the traumatic event (2). Research suggests behavioral responses to trauma (e.g., avoidance, hyperarousal), relative to internalized symptoms (e.g., a sense of a foreshortened future or self-blame), are more common or at least more observable in young children and individuals with significant developmental delays (3, 4). These developmental differences in traumatic responses have prompted the adoption of adjusted assessment approaches and PTSD criteria for these groups (3, 4).

In response to trauma, people attempt to cope. Coping is a broad term encompassing cognitive and behavioral strategies that vary across individuals and situations, but share a common purpose “to manage specific external and/or internal demands that are appraised as taxing or exceeding the resources of the person” (5). Coping has been found to moderate the development of PTSD: for example, coping centered around avoidance or self-blame may strengthen relationships between trauma exposure and traumatic stress, whereas coping focused on positive reappraisal may weaken this link (6, 7). Similarly, avoidant coping strategies have been associated with increased PTSD symptomology (8, 9) and poorer PTSD treatment response (10). Extensive literature on the post-traumatic coping of non-autistic individuals has informed several different coping conceptualizations and measures (11). One widely-cited framework, proposed by Tobin et al. (12), that we used for this study, situates coping strategies along two dimensions – problem vs. emotion focused, and approach vs. avoidance focused. Though avoidance-focused strategies are traditionally viewed as maladaptive, and approach-focused strategies as adaptive (11), this dichotomy has recently been challenged by research demonstrating that the functionality of coping is nuanced and context-dependent (13, 14). For example, avoidance strategies may serve a protective function for children facing urgent and uncontrollable stress; however, overreliance on avoidance outside of these situations prevents children from learning alternative coping skills, and over time, avoidance behaviors may place them at risk for developing mental health difficulties, including PTSD (15, 16).

As with traumatic reactions, it has been suggested that coping behaviors may vary with one's developmental level: although children can and do employ similar strategies to adults, there is a tendency for people to adopt more problem-focused (i.e., directly attempting to alter the source of trauma), and fewer avoidance-based (i.e., attempting to circumvent situations that provoke trauma-related symptoms) strategies as they age (17). Elsewhere, developmental theories of coping posit that the adoption, specificity, and effectiveness of problem-focused coping coincides with neurological development and the emergence of increasingly sophisticated executive functioning abilities, such as cognitive flexibility and fluid reasoning (15, 18). Other research examining how adults with intellectual disability cope with social stress suggests that their coping strategies largely align with those used by the general population (19), but may be focused more on avoiding trauma-associated situations than on gaining control over a situation (19, 20). Further, within active coping, strategies focused on altering the source of trauma appear to be more readily used than those focused on altering one's emotional state (19). In sum, the literature on coping in different populations suggests that individual differences (e.g., developmental level, intellectual ability) play an important role in shaping the coping strategies available to different people.

Autistic individuals experience higher rates of potentially traumatic events [PTE; (21–23)] than the general population. These events have been linked to elevated rates of mental health conditions in autistic youth (24, 25) and PTSD in autistic adults (26). Researchers have proposed transactional relationships in which autistic traits (e.g., social communication differences, patterns of restricted and repetitive behaviors and interests, sensory sensitivities) and behaviors related, but not central to, an autism diagnosis (e.g., emotion dysregulation) influence the experience of trauma at multiple levels – including what stressful events are encountered and experienced as traumatic, and how traumatic reactions develop and manifest (26–29). These ideas are supported by studies suggesting elevated rates of PTE (22, 23, 30–32), PTSD and other traumatic reactions (26, 28, 33, 34) among autistic people.

Coping may be a crucial mechanism linking PTE to traumatic reactions for autistic people. It has been hypothesized that autistic individuals may struggle to cope effectively with PTE, perhaps due to increased social marginalization, and executive functioning and emotion regulation challenges (26, 27, 29). Moreover, researchers have suggested that traumatic reactions may increase autistic traits via coping behaviors, suggesting a complex relationship between these conditions (27, 35). Empirical research in non-trauma contexts suggests that autistic children use more avoidance and venting strategies, and fewer emotion regulation and social support strategies, compared to their non-autistic peers (36, 37). Recently, qualitative research has begun to explore autistic people's experiences of coping in everyday life. Studies have found that autistic adults report a wide range of strategies or experiences that help them cope and build resilience, including strategies commonly reported in the broader coping literature, such as engaging in recreational activities or seeking social support, as well as strategies that may be more autism-specific, such as seeking support from the autistic community, using technology to facilitate social interactions and daily activities, masking, or self-understanding and acceptance following their autism diagnosis; (38, 39). Elsewhere, autistic adolescents and adults have described stimming as an adaptive coping mechanism that helps them self-regulate, self-soothe, and communicate when faced with overwhelming sensory environments, thoughts and emotions, or uncertainty (40, 41). Taken together, an emerging literature suggests that autistic people engage in a variety of coping behaviors, some of which converge with existing categorizations and definitions of coping, and some of which may be specific to autism. However, limited research to date has focused on autistic people's experiences of coping with trauma specifically. Moreover, existing research has focused primarily on autistic adults' first-hand narratives. Parental perspectives may provide valuable information on how autistic children, or those with varied communication abilities, respond to trauma. Although past qualitative studies have shed light on how the parents of autistic individuals cope with stressors (42), research has yet to ask the parents of autistic individuals about their children's coping behaviors.

Research suggests that what constitutes a traumatic event and reaction varies across autistic and non-autistic individuals (27, 29, 43–45). In the same way, coping may also vary between autistic and non-autistic people. An important step toward understanding how autistic people respond to trauma is to conduct open-ended explorations of diverse personal narratives. This may help generate accurate frameworks for future research and practice. Therefore, in this study we used qualitative methods to explore firsthand and caregiver perspectives on how autistic people cope with trauma, with the aim of describing the range of coping strategies used by autistic individuals.

## Methods

### Participants

Participants took part in a broader study of the sources, nature, and experiences of trauma among autistic people (46). The current study is a secondary analysis of the data collected in this original study. In total, fourteen autistic adults and 15 caregivers of autistic individuals (hereafter collectively referred to as “participants”) were recruited via US-based autism-related organizations, the researchers' professional networks, or word of mouth. Using stratified purposive sampling, participants of varied genders, ages, clinical presentations, and adversity histories were included to ensure a diverse range of perspectives. Participants were required to be 18–70 years old and fluent in English. Caregivers (biological or adoptive parents) were selected to ensure representation of individuals unable to participate themselves (i.e., very young children or individuals with significant communication challenges). Three parent-child dyads were included in the final sample. Upon enrolling in the study, participants completed a study-specific survey of socio-demographics, diagnostic history, and for parents, their child's communication ability. Participants also completed measures of PTE [Trauma History Questionnaire [THQ; (47, 48)]] and, for autistic adults, a self-report of post-traumatic symptoms [PTSD Diagnostic Scale for adults [PDS-5; (49)]].

Autistic adult informants (Table 1) were predominantly Caucasian (*n* = 11; 79%) and male (*n* = 9; 64%). The majority (*n* = 11; 79%) had an annual income of < $25,000. All 14 were verbally fluent. Caregiver informants (Table 2) were predominantly mothers (*n* = 12; 93%), and more racially and socioeconomically diverse. The majority of caregiver informants' children were male (*n* = 13; 87%) and had some degree of difficulty communicating (*n* = 11; 73%). Details of psychiatric comorbidities (per self-reported community diagnoses) in the sample are provided in Tables 1, 2. Anxiety, depression and OCD were the most common comorbidities. Community diagnoses of PTSD were reported by three (21%) individuals in the autistic adult sample and two (13%) caregivers, both of adult children. Among the autistic adult participants, 10 (71%) scored above the clinical cut-off on the PDS. These results are consistent with prior work suggesting that PTSD is often undetected and undiagnosed in autistic people (26); however, the research examining the accuracy of the PDS in autistic samples is also needed.

**Table 1:** Characteristics of autistic adults.

**Adult informants M(SD) or %(*****N*****);** ***N*** **=** **14**			
**Age** Age Range	37.14 (12.53) 22–64 years	**Traumatic stress symptoms**	
		PDS-5	35.57 (17.61)
**Gender**		**Prior diagnoses**	
Male Female Transgender	64.29% (9) 28.57% (4) 7.14% (1)	PTSD Anxiety Depression	21.43% (3) 64.29% (9) 35.71% (5)
**Race** Caucasian	78.57% (11)	Bipolar/Mood disorder	7.14% (1)
Asian	7.14% (1)	ADHD	28.57% (4)
Biracial	14.29% (2)	OCD	42.86% (6)
**Education**			
2-year Degree/Some College College Degree Graduate Degree	35.71% (5) 42.86% (6) 21.43% (3)		
**Annual income**			
< $25,000 $25,001–$50,000 $50,001–$75,000	78.57% (11) 14.29% (2) 7.14% (1)		
**Environment**			
Rural Suburban Urban	7.14% (1) 50.00% (7) 40.00% (6)		

**Table 2:** Characteristics of parent informants and their children.

**Parent informants M(SD) or %(*****N*****);** ***N*** **=** **15**			
**Parents**		**Children**	
**Age**	45.92 (10.01)	**Age** Age Range	16.47 (7.91) 5–29 years
**Relationship to child**		**Gender**	
Father Mothers Couples	6.67% (1) 80% (12) 13.33% (2)	Male Female	86.67% (13) 13.33% (2)
**Race**		**Communication ability**	
Caucasian Black/African American Asian Hispanic	66.67% (10) 20% (3) 6.7% (1) 6.7% (1)	No trouble communicating Little trouble communicating Lot of trouble communicating	26.7% (4) 33.3% (5) 40.0% (6)
**Education**		**Prior diagnoses**	
2-year Degree/Some College College Degree Graduate Degree	33.33% (5) 33.33% (5) 33.33% (5)	PTSD Anxiety Depression Intellectual Disability	13.33% (2) 60% (9) 33.33% (5) 6.7% (1)
**Annual income**		***ADHD***	26.7% (4)
< $25,000 $25,001–$50,000 $50,001–$75,000 >$75,000	26.7% (4) 6.7% (1) 13.3% (2) 53.3% (8)	Behavior/Conduct Problems OCD	20% (3) 40% (6)
**Environment**			
Rural Suburban Urban	6.7% (1) 40% (6) 53.33% (8)		

Autistic adults and the children of participating caregivers needed to have (a) received a community diagnosis of autism and (b) experienced a self-reported traumatic event that negatively impacted their well-being for >1 month (per self/parent report), a time-criterion commonly used to differentiate acute from post-traumatic stress (2). Autism status was corroborated by ensuring all participants exceeded clinical cut-offs for autism on either the Ritvo Autism and Asperger Diagnostic Scale-Revised Screen [RAADS-14; clinical cut-off >13; (50)] for adults, or the Social Communication Questionnaire [SCQ; clinical cut-off >15; (51)] for children. Given studies suggesting that for autistic individuals, traumatic reactions arise from a broad array of experiences and not only those events defined in PTSD criteria (28, 46, 52), trauma sources were not restricted so long as they were associated with prolonged (>1 month) distress. Trauma sources, as reported by participants via the THQ and qualitative interviews, are presented in Supplementary Material 1: physical abuse, emotional abuse, bullying, and “other traumas,” which included varied forms of social exclusion, instances of sensory overload, and traumatic transitions/changes [see (46), for further information].

### Interview Procedure

Procedures were approved by the Drexel University Institutional Review Board. Participants gave informed consent. Data was collected during 60–90 min qualitative interviews, conducted and recorded by CMK – a Caucasian female, clinical psychologist, and researcher with expertise in autism and stress-related disorders. Interviews were completed in-person at the research center (*n* = 13), over the phone (*n* = 15), or via email (*n* = 1) according to participant preference over a 6-month period in 2017. During the interview conducted via email, the interviewer sent all interview questions to the participant in a single document. Responses, once returned via email, were reviewed by the interviewer, who then sent a final round of follow-up questions to clarify meaning as needed.

The interview guide is provided in Supplementary Material 2. The guide was developed with a multidisciplinary (social work, psychology, psychiatry, epidemiology, sociology) team and revised per stakeholder (autistic adults, caregivers) recommendations, to ensure sensitivity and offset researcher positionality. As autistic individuals may experience a wider range of events as traumatic than defined in the Diagnostic and Statistical Manual (DSM-5; 2), participants were given the SAMHSA (1) trauma definition (previously outlined in the Introduction) and asked to describe events in their/their child's life they perceived as traumatic. They were also asked to describe coping strategies: all participants were asked, “Were there things you/they did to help yourself/themselves feel better or to get through?” Field notes taken during interviews were shared with participants for clarification and validation [for further protocol details see (46)].

### Thematic Analysis

**Figure 1:**
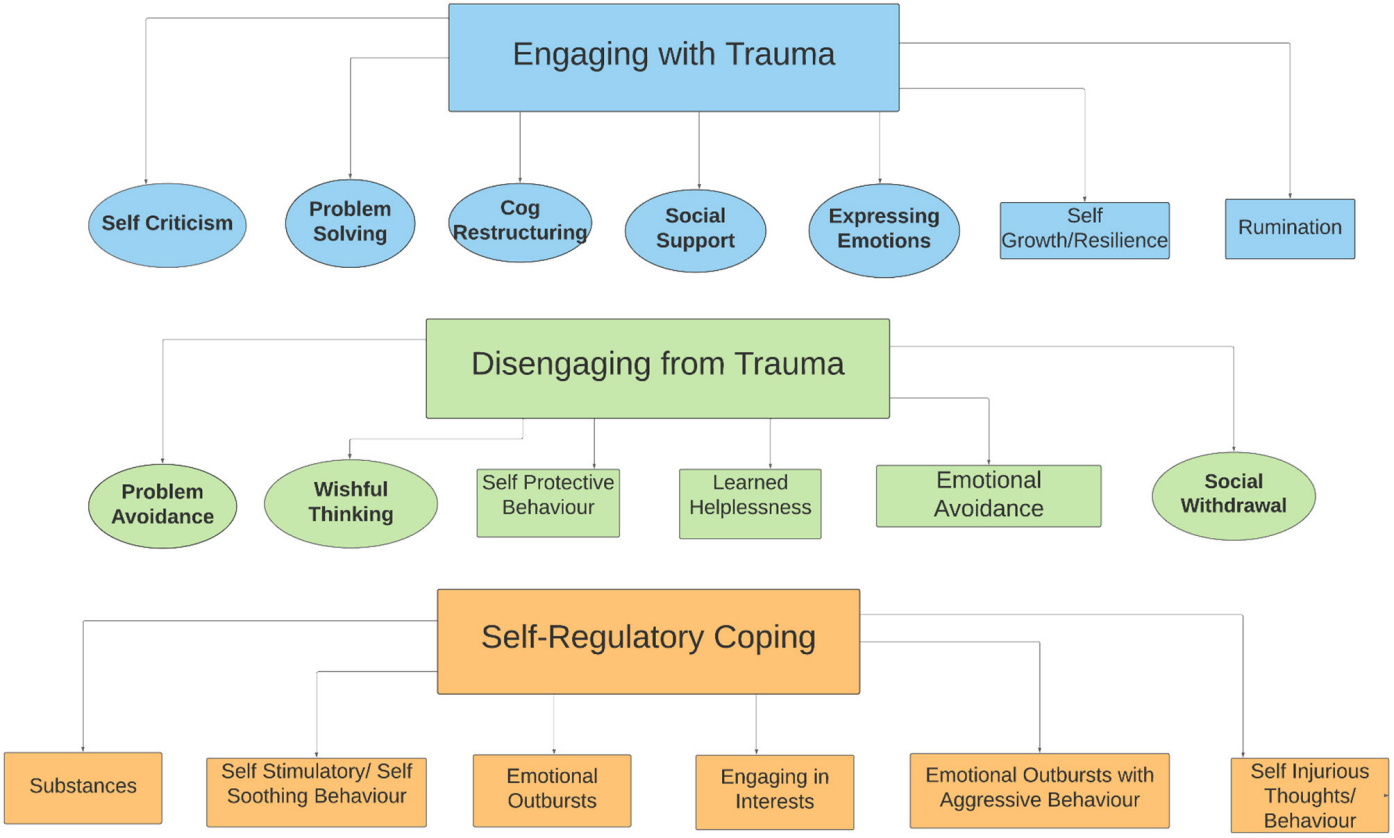
Thematic map of coping strategies. Circles represent deductive codes [originating from Tobin et al. (12) framework]; rectangles represent inductive codes [any codes identified in dataset not outlined in the Tobin et al. (12) framework]; green codes indicate the Disengaging from Trauma theme, blue codes the Engaging with Trauma theme, and orange codes the Self-Regulatory theme. This figure was created in Lucidchart.

Interviews were transcribed verbatim and submitted to thematic analysis using Nvivo (53). All participants were assigned a pseudonym. Using a realist framework and semantic coding approach, which integrated inductive (a ‘bottom-up' approach whereby themes are generated from data, not pre-existing theory) and deductive (a ‘top-down' approach; driven by prior theory) analyses, themes were identified via six iterative stages: data familiarization, coding, theme generation, review, and definition, and writing (54, 55). This approach allowed the identification of coping strategies already prominent in the existing coping literature (deductive), as well as behaviors less commonly identified as coping, or potentially more specific to autism (inductive). Deductive codes and identification of themes were guided by Tobin et al.'s (12) theoretical framework, but then modified to include inductive codes/themes (see Figure 1).

Two primary analysts (AR and HP) familiarized themselves with four interviews (two adult, two caregiver), and identified codes via line-by-line readings and discussion (56). A resulting coding scheme was refined with input from other team members (ENC and CMK), then applied to another four interviews (two adult, two caregiver), revised and formalized. Analysts applied this coding scheme to all remaining interviews. The broader coding team met regularly to resolve discrepancies and revise themes iteratively and reflexively. Inter-rater reliability was calculated (prior to resolutions) using percentage agreement (#inter-rater agreements / total #extracts coded; mean = 96%; range = 92–100%) or, for low prevalence codes (present in <14 interviews; *n* = 7), prevalence-adjusted, bias-adjusted kappa [mean = 0.96, range = 0.86–1.00; (57)]. The final set of themes and subthemes was organized and refined based on discussions with all co-authors. The authorship team comprised women and men of Caucasian, East Asian and South Asian descent, at early, mid, and senior career levels, with training and expertise in psychology, psychiatry, sociology, social work and epidemiology, as well as experience being on, or caring for someone on the autism spectrum.

## Results

We identified 19 subthemes (each representing a coping strategy), three main themes, and one integrative theme (Figure 1, Table 3). Throughout this section, the term “participants” refers collectively to autistic adult and caregiver informants. More specific language is used to denote instances in which observations pertain primarily or solely to one group (e.g., autistic adults but not caregivers).

**Table 3:** Themes and subthemes.

**Theme/Subtheme**	**Frequency (*N*)**	**Definition**	**Inductive/ deductive**	**Examples**
**Engaging with trauma**				
Problem solving	Adults = 11 Parents = 7	Taking concrete steps or direct action to address or alleviate sources of stress	Deductive	“*In my marriage I started out running to my parents every time me and my wife fought over something, but now I try to solve it myself…That's the hardest thing for people on the spectrum is being their own problem solver.”* (Jasper, 27-year-old man)
				“*…Two years ago … he came upstairs and said ‘I just went on Facebook and here's this post from my sister saying she's homeless and begging for places to live- I can't take this anymore, I'm blocking them on Facebook cause I have a degree I need to get and I can't look at this' and that was the first time I've ever really seen him proactively saying ‘there's more coming and I just won't be part of it'.”* (Marissa and Tom, stepparents of 22-year-old man, Josh)
Social support	Adults = 14 Parents= 15	Utilizing others for assistance or support	Deductive	“*I have one really close friend … we're like sisters but she lives in England and I'm thankful for email because that was where a lot of it* [stress over threat of homelessness] *went, I knew that she understood.”* (Jenny, 64-year-old woman)
				“*…he was calling me like every hour… he just had a really hard time not understanding why he was being left out… And he will call me and tell me how lonely he is.”* (Karen, mother of 20-year-old man, Justin)
Expressing emotions	Adults = 4 Parents = 8	Explicitly communicating one's emotional state	Deductive	“*…This last school year she's been having more anger and we've talked about the fact that she's autistic and she can't speak so she's been displaying a lot of frustration with the fact that she's autistic because she can type, so she says ‘I hate being autistic', ‘I hate being me', ‘I want to be able to talk', ‘I wish I could be like my sister'.”* (Samara, mother of 9-year-old girl, Zahara, who is non-verbal but communicates through typing)
				“*I'll go off into another room just to think about what actually I want to say and how I want to express my anger… I've got to take a step back, take a deep breath, leave the room, and* [tell my wife]*, ‘I am angry with you because you said this'.”* (Jasper, 27-year-old man)
Self-criticism	Adults = 5 Parents = 7	Blaming or criticizing oneself for the trauma	Deductive	“*I still have this fear that I'm going to do something wrong at my job, that one little thing. Cause there were parts at my current job in which… I forgot to do one thing and it sort of screwed everything up and that got me all anxious and angry at myself.”* (Randy, 27-year-old man)
				“*…He would sometimes ask me to send him away. ‘I'm terrible, I'm horrible, I hit you, I did this to you, I did that to you, you should just send me away, send me to an orphanage.' …He catastrophizes everything, like everything is the worst day ever, ‘I'm the worst kid ever'.”* (Dorothy, mother of 12-year-old boy, Riley)
Cognitive restructuring	Adults = 10 Parents = 2	Actively attempting to change thought patterns that may be negative/ harmful, looking at things from different perspective	Deductive	“…[I] *try to think positive thoughts about myself, my friends, and even some positive thoughts about the people in my environment that have been bothering me. If I think some positive thoughts about them then that may actually help me and it has.”* (Alex, 37-year-old man)
Rumination	Adults = 9 Parents = 7	Repeatedly experiencing negative thought patterns in relation to the trauma/stressor	Inductive	“*I didn't recover from stressful or upsetting events the way normal children did. I didn't have a normal ability to regulate my emotions, and I couldn't calm myself down the way other children my age could, but because of my age the adults around me decided I should be able to and refused to help me… If I do something wrong or if someone does something bad to me, it never, ever goes away. It just replays in my mind at intervals for the rest of my life. Not constantly, but it just keeps coming back…”* (Martha, 37-year-old woman)
				“*And she still dwells on it as she dwells on a lot of the other traumatic experiences… It means a lot of talking to herself at night, she goes through long lectures to herself, long discussions with other people and so on when they're not there.”* (Ken, father of 29-year-old woman, Amal)
Self-growth/resilience	Adults = 11 Parents = 8	Transforming past trauma into positive learning experiences or	Inductive	“*I give people like me more latitude with behavior because I know it's difficult.”* (Darrell, 52-year-old man)
		opportunities for personal growth/self-awareness		“*He is a resilient kid, he has really done the work of establishing that relationship* [with his father] *and he's done the work of being forgiving … so this poor kid got kicked to the curb but he's very forgiving…”* (Tina, mother of 16-year-old boy, John)
**Disengaging with trauma**				
Problem avoidance	Adults = 6 Parents = 6	Actively avoiding the problem or the stressor	Deductive	“*…He would you know start warming up to the school again, and then* [being secluded or physically restrained] *would happen… then the whole year would be ruined because then he would refuse to go to school… and it would just…go right downhill”* (Akira, mother of 22-year-old man, Malik)
				“*I run away… try to avoid her* [mother who has been emotionally abusive] *as much as possible. Ignore her constant phone messages and texts and stuff.”* (Antonio, 24-year-old man)
Social withdrawal	Adults = 9 Parents = 8	Withdrawing from social relationships and/or avoiding social	Deductive	“*I decided it's easier to be a loner than it is to try to fit in where I don't belong.”* (Peter, 39-year-old man)
		situations		“*I said it is trauma because I think he shied away from people after [being bullied] and he really doesn't have any friends and doesn't really connect with people. And I know part of the that is the Asperger's, but there are people with Asperger's that do have friends.”* (Rihanna, mother of 26-year-old man, Paz)
Wishful thinking	Adults = 5 Parents = 2	Engaging in fantasy to avoid dealing with the stressor in hopes that	Deductive	“*As a kid I would fantasize about running away.”* (Martha, 37-year-old woman)
		the problem will go away		“*He doesn't want to grow up basically… when they talk about jobs and pay and growing up and being independent, I don't think that he likes that. He wants to remain a child and one of the reasons I think is that when he was a child he had a family, I think that losing the family was the biggest traumatic event for him and he still wants to remain a child I think.”* (Anita, mother of 26-year-old man, Sam)
Emotional Avoidance	Adults = 7 Parents = 2	Concealing or suppressing emotions in order to keep functioning	Inductive	“*It's almost like I was catatonic* [after the death of my friend]. *I stopped feeling anything all together.”* (Peter, 39-year-old man)
				“*I think he can really compartmentalize. He can really stuff things down and keep going as though it didn't happen because he's had no choice…but he's paid for that in being told ‘you have no feelings', ‘you're robotic', but otherwise, I don't think he would have survived, I'm not sure.”* (Marissa and Tom, stepparents of 22-year-old man, Josh)
Learned helplessness	Adults = 11 Parents = 5	Adopting the perspective that the situation will never change and/or feeling	Inductive	“*I had no one to go to… no one's coming to save me, no one's bailing me out, there's no superman to the rescue.”* (Malik, 22-year-old man)
		hopeless		“*…When he's feeling sad he uses it as a ‘oh well I have autism there's no hope for me… I don't need to worry about school because I'm never going to be able to be anything'…”* (Dorothy, mother of 12-year-old boy, Riley)
Self-protective behavior	Adults = 10 Parents = 6	Being cautious in certain situations due to past trauma	Inductive	“*I learned pretty early on it's not always a good thing if you attract the attention of adults so I kind of learned how to fly below the radar”* (Jenny, 64-year-old woman)
				“*He is very vigilant* [after being physically assaulted by school staff and peers]…* No matter who it is. His trust level is like zero.”* (Akira, mother of 22-year-old man, Malik)
**Self-regulatory coping**				
Substances	Adults = 7 Parents = 7	Utilizing substances to cope with trauma	Inductive	“*I abused substances a lot. Like when I was going out with* [partner]*, I drank a lot and wanted to get high on everything I could put my hands on. Just basically to escape how dysfunctional the relationship was.”* (Arya, 38-year-old woman)
				“*I think, this is my personal opinion and I don't know if this is why he started smoking, it was so he could stand with smokers… It was a social thing, he was trying to fit in. And then he got hooked to the cigarettes.”* (Rihanna, mother of 26 year old man, Paz)
Engaging in interests	Adults = 11 Parents = 12	Participating in hobbies or activities to cope	Inductive	“*…We'd have probably a half a library's worth of books, not even joking, we were avid readers … he's had the same night routine since, well honestly before he was born. I think* [reading] *helped with the moving.”* (Georgie, mother of 6-year-old boy, Timmy)
				“*There was no place I could go that was quiet, there was no place you know eventually I had a room of my own in the house and I started accumulating stuff… I think I was born to be a collector”* (Lina, 53-year-old woman)
Self-injurious thoughts/behaviors	Adults = 11 Parents = 9	Redirecting feelings to imagine escape from stressor or trauma	Inductive	“*He is deathly afraid of fire drills and loud sounds startle him… And he told me he was, he actually made a suicide threat. He said I'll kill myself, I didn't realize in the moment it was about a fire drill, but he picked up a drill like a cordless drill and held it to his head, and said I'll kill myself I'm not going to school. I told him to put the drill down, and he did, and I kept him home that next day.”* (Dorothy, mother of 12-year-old boy, Riley)
				“*My father had just bought another house and my mother said well I'm not moving there, and that was it. And that was how it happened. And I recognize that, you know, that's where the emergence of the skin picking and scratching happened and um, you know, it was to a point where I had sores all over my skin… That wasn't something I knew then and as I kid had no idea that had something to do with my parents getting divorced. There was no way I was able to connect those as issues. But now have a lot of understanding of those things being related.”* (Charlie, 22-year-old man)
Self-stimulatory/self-soothing behavior	Adults = 7 Parents = 11	Behaviors the participant engages in to calm themselves or self-regulate, usually in overwhelming situations	Inductive	“*There's certain things that make her feel better in general when she's anxious and tense* [and possibly after her grandfather died] *and they're kind of stimmy behaviors. Like her necklaces… the beads that they put in the bottom of the vase, the glass, her bins are full of those- she loves to run her hands through those.”* (Samara, mother of 9-year-old girl, Zahara, who is non-verbal but communicates through typing)
				“*…During the time that I'm* [brushing my skin with a neck brush]*, it has a sound that it makes and when I have brushed on part of the skin and I start brushing somewhere else, there's still like a shadow of the sensations of where it had been and… listening to it and feeling that sort of tracking- that is something that blocks out any of the mental processes that are happening- it's grounding.”* (Charlie, 22-year-old man)
Emotional outbursts without aggressive behavior	Adults = 3 Parents = 11	Situations where the participant experiences overwhelming emotions, resulting in an outburst	Inductive	“*I couldn't deal with all this anger and all this hate and all this frustration inside* [from bullying, physical abuse by peers, and repeatedly transferring to new schools]*, I couldn't handle it, I couldn't keep it in, and… it came out in yes, screaming… it came out in meltdowns…”* (Malik, 22-year-old man)
Emotional outbursts with aggressive behavior	Adults = 7 Parents = 8	Emotional outbursts that are accompanied by aggressive behavior toward another person or object		“*All of the behaviors are associated with that anxiety and his lowering of his self-esteem but… when he was upset he would just punch the wall or like many times he has punched me and his dad and…”* (Layla, mother of 19-year-old man, Sameer, who is minimally verbal)
**Diagnostic overshadowing**				
	Adults = 8 Parents = 14	Instances in which coping behaviors overlap with, or are confused for, autism-related behaviors	Integrative	“*I don't want to give anyone any information that might be used against me, and I think that people just are quick to look at* [me] *like, ‘something's wrong with him but I'm not sure what, something about him rubs me the wrong way', but they don't think maybe of the history…”* (Malik, 22-year-old man)
				“*A lot of behaviors* [e.g., meltdowns] *that…if you saw a neurotypical child showing, most people… would say something is not right there, whereas with autistic children, sometimes it might mean that something is very wrong and it sometimes it may just be their normal autistic behavior”* (Georgia, mother of six-year-old boy, Timmy)

### Engaging With Trauma

This theme encompassed seven subthemes in which participants actively addressed or managed trauma-related thoughts, emotions, or situations. Regarding deductive codes, *problem-solving* involved taking concrete steps to mitigate a traumatic situation: autistic adults reported setting interpersonal boundaries, for example with bullies or within toxic relationships. Parents noted children used problem-solving to navigate interpersonal problems and sensory situations, such as informing parents of overstimulating situations in advance. Both children and adults sought others' help to solve problems, such as reporting a situation to the school principal, or engaged in the problem more directly, for example proactively blocking a family member on social media to prevent further distress and distraction from one's studies. However, children tended to problem-solve by seeking others' help, whereas adults tended to engage with the problem more directly. *Social support* was the most commonly endorsed coping strategy, with every participant noting that autistic individuals relied on others for support in at least one traumatic situation. Support came from many sources, including family, friends, therapists, doctors, teachers, and pets; moreover, the types of support also varied. Some participants received practical support to complete specific tasks, such as assistance from a job coach in navigating the stressful process of job interviews. Others sought out emotional support, such as physical and emotional comfort from friends, family, and pets. Martha, a 37-year-old woman, spoke of the bond she had with her bird, who was “one of my reasons for living.” Children tended to receive support from family members (primarily parents) but also sought from friends, professionals such as a school counselor, social groups, and members of the community, such as neighbors or a librarian. Adults most often received support from professionals such as a job coach, lawyer, psychiatrist, or social worker, although they also reported being supported by friends and family. Participants also described *expressing emotions*, which entailed autistic people sharing trauma-related emotions with others as an outlet to process their trauma, rather than seeking practical support to address the traumatic situation itself. Adults and children alike mostly turned to family members, like parents, grandparents, or partners to express their emotions. However, adults also turned to professionals such as a job coach, or in one case, expressed their emotions to themselves. Participants noted both social support and expressing emotions had positive implications, especially when support came from those close to them, like parents, friends, or teachers.

Autistic individuals also engaged in *self-criticism –* blaming themselves (as opposed to their environment or circumstances) when attempting to understand the cause of their trauma and distress. Autistic adults attributed interpersonal trauma such as social exclusion or bullying to themselves. Parents described their children blaming themselves for their communication differences and for feeling “different” or disconnected from others. Several autistic adults also blamed themselves for encountering and reacting to abuse. Martha not only blamed herself for her sexual assault, but had also internalized the idea that sexual assault of women is socially accepted: “I felt so ashamed...and I felt stupid for getting upset at all because I know this happens to all women and nobody else lets it bother them.” Participants also described *cognitive restructuring*, or actively changing thought patterns to reframe traumatic experiences. This strategy was largely used by autistic adults, who described it as a way to reflect on past experiences with self-compassion. Some participants reported that cognitive restructuring occurred after learning that they were autistic provided a new perspective on their experiences. Others reported that friends, partners, job coaches, social workers, and therapists had helped, or directly taught them to reframe their experiences. Cognitive restructuring was largely used by adults; although one participant recalled that receiving their autism diagnosis as a child had helped them reframe their traumatic experiences.

Regarding inductive coping themes, *rumination* (repeated negative thoughts about the trauma) was noted by both parents, who described their children constantly worrying about past and anticipated sensory stimuli such as thunderstorms and haircuts, and autistic adults, who described replaying past interpersonal traumas. Although sometimes described as an attempt to understand trauma, rumination, similar to self-criticism, was typically associated with prolonged psychological distress for autistic adults and children. Lastly, participants reported *self-growth/resilience* (transforming past trauma into opportunities for growth or self-awareness), which typically occurred after a positive turning point, like meeting a spouse or a group of friends. Some autistic adults reported using past trauma to help others in similar situations; others described developing empathy toward others with social challenges. Adults and children found self-growth/resilience through the process of receiving their autism diagnosis, getting help from professionals such as therapists or counselors, and independent choices and actions, for example by deciding to forgive people who had hurt them in the past, or finding a purpose in life. Ultimately, self-growth/resilience captured positivity and meaning that could derive from traumatic experiences.

### Disengaging From Trauma

This theme encompassed six subthemes involving autistic individuals withdrawing from trauma-related emotions, thoughts, or situations. Regarding deductive themes, several autistic adults and parents of autistic children reported *problem avoidance –* avoiding physical places, activities, or situations directly related to the trauma (e.g., staying away from school to avoid bullies). Both children and adults reported problem avoidance; however, this coping strategy was more often reported by children. Children largely reported avoiding places that were associated with their trauma, like home or school. Relatedly, participants described *social withdrawal* as a specific attempt for autistic people to distance themselves from others socially, in order to protect themselves from further challenges navigating social situations, social exclusion, or victimization. Participants also described nuanced forms of disengagement such as *wishful thinkin*g, in which autistic individuals used fantasy or daydreaming to escape trauma-related distress. Some autistic adults reported thinking of ways they could have fought back against bullies, or wishing they were born into a different life. Several parents described children engaging in wishful thinking about their autistic identity: Samara recalled her nine-year-old daughter telling her, “I wish I was like my [non-autistic] sister.”

Regarding inductive themes, participants described *emotional avoidance –* concealing and/or suppressing one's emotions. Autistic adults reported suppressing their emotions both internally (e.g., disconnecting from their pain, feeling numb) and outwardly (e.g., hiding their emotions, appearing “blank”) after receiving negative feedback from others for expressing their emotions in the past. Parents of younger children described their children ‘shutting down' and hiding their distress. Participants also described *learned helplessness*, in which autistic individuals adopted a hopeless mindset in relation to their trauma, in order to prevent continued feelings of being let down or disappointed. This often occurred when they felt their situation was beyond their control and would not change, such as being socially marginalized. A sense of helplessness was often described after repeated abuse or interpersonal betrayal. Learned helplessness was more often used by adults and older children after repeated and chronic traumas like prolonged bullying. Lastly, participants described *self-protective behavior*: remaining vigilant to avoid repeating past traumas. For example, one individual described carrying a backpack of personal items after being stranded as a child, while another described being wary of adults following childhood abuse by caregivers. As with learned helplessness, autistic adults and older children more commonly engaged in self-protective behaviors after repeated or chronic traumas.

### Self-Regulatory Coping

This theme included six inductive subthemes involving attempts to calm oneself after experiencing a stressor. While some of these strategies showed potential overlap with *Disengaging From Trauma*, they were also described as attempts to manage internal trauma-related distress rather than, or in addition to the stressor itself. Some autistic individuals coped with *substances*, such as alcohol, food, and drugs. With the exception of prescribed medication, which was used by both adults and children, substances were largely used by adults and served varied functions. Participants noted marijuana and prescribed medication helped bring trauma-related thoughts, emotions or stress down to more manageable levels. On the other hand, illicit drugs and alcohol were typically described as a way to escape or numb distress. Participants described *engaging in interests* (e.g., music, writing, electronics) as a way to immerse oneself in activities that could bring joy, calm, and distraction from a stressor. Children engaged in interests such as video games, listening to music, television shows, and physical activity such as gymnastics, while adults reported engaging in hobbies such as attending science fiction conventions, writing, art, and dressing in costume. *Self-injurious thoughts and behaviors* were described by parents and autistic adults as a way for autistic individuals to channel trauma-related emotions into physical action after reaching a breaking point. For example, parents speculated their child would bang their head when overwhelmed with emotional or sensory pain. Autistic adults also described cutting and burning themselves when they became overwhelmed by stressful situations, and engaging in suicidal thoughts to provide hope for an escape from their pain, and therefore temporary relief, when feeling despondent about their circumstances. Children tended to engage in self-injurious behaviors like head banging, or scratching and hitting themselves, whereas adults were more likely to engage in suicidal thoughts or attempts. Autistic adults reported, and parents speculated, that *self-stimulating/self-soothing behaviors*, such as rocking, pacing, and repetitive movements, helped autistic individuals calm themselves when distressed. Both children and adults engaged in stimming, although it was more common among children. Other forms of self-stimulating/self-soothing behaviors used by both adults and children included using sensory items such as beads or a weighted blanket, or finding a safe space/quiet room to decompress. Participants also noted *emotional outbursts (with or without aggressive behavior)*, most often described as meltdowns, as a way to release overwhelming distress in traumatic situations. Situations ranged from sensory overstimulation, like getting a haircut, to interpersonal traumas, such as bullying. Emotional outbursts were largely used by children of varied ages and communicative abilities.

### Diagnostic Overshadowing

Through the coding process, we observed that participants described overlap (and often confusion) between coping and autistic traits or autism-related behaviors. We therefore developed an integrative theme, Diagnostic Overshadowing, to highlight this issue, which was relevant to coping behaviors from the Disengaging from Trauma (*social withdrawal, emotional avoidance)* and Self-Regulatory Coping (*emotional outbursts, self-injurious thoughts and behaviors, self-stimulatory/self-soothing behaviors)* themes. Parents and autistic adults alike recalled the challenges arising from this overlap, including instances in which signs of trauma had been overlooked because they had been mislabelled as autism-related behaviors. For example, when describing her 5-year-old son's increasing social withdrawal following several traumatic hospitalisations, Maria stated, “I don't know if it's related to the trauma or related to just understanding that he's autistic.” Elsewhere, when discussing her 11-year-old son's emotional outbursts after experiencing abuse, Raisa noted, “I don't know what is the autism and what is the trauma…he already has autism but I feel like everything just quadrupled, like the reactions, the sensitivities.” Raisa also described the serious implications of this ambiguity for court proceedings:

“…*it was like a double-edged sword–being autistic. You would think that they would…try to understand that part of him more, but instead they would try to use it like ‘oh no, that reaction is from the autism, it's not from the trauma'.”*

Autistic adults also described how others could not only fail to recognize signs of trauma and coping, but could also view coping strategies (e.g., *self-stimulatory/self-soothing behaviors*) as problematic and needing to be “fixed”. Martha noted

“*I think a big part of it is that we're already known for behaving oddly, and we're often in some sort of therapy or behavior modification to address it, so people are not expecting us to act normally. There's also a mindset of “fixing behavior problems”, so I think signs of distress or trauma are approached as just another irritating habit we need to be broken of, and nobody stops to think about why we're doing it.”*

## Discussion

This study interviewed verbally fluent autistic adults and caregivers of autistic children of varying ages and adaptive abilities to explore the varied ways in which autistic individuals cope with traumatic experiences, including but not limited to stressors outlined in PTSD criteria (e.g., abuse, life-threatening accident). To examine this research question from multiple angles and illuminate common themes, individuals with diverse experiences and characteristics were purposefully recruited.

We found that autistic people use multiple and varied strategies to cope with trauma, showcasing the multifaceted and flexible nature of coping (58). During interviews, participants described strategies within three main themes, largely consistent with existing theories of coping: Engaging with Trauma, Disengaging from Trauma, and Self-Regulatory Coping. A key component of coping frameworks is the distinction between approach vs. avoidance (11, 12), which aligns with our themes of engagement and disengagement. Interestingly, self-regulation has been proposed to cover a broad set of skills cutting across cognitive, emotional, and physiological domains, and to overlap with coping (59). Indeed, the Self-Regulatory Coping theme encompassed behaviors that could be framed as forms of disengagement (e.g., *substances, engaging in interests, and self-injury*), yet were consistently described by participants as autistic individuals' attempts to modulate internal thoughts or emotions. To capture and reflect these perspectives, we conceptualized these behaviors as serving a primarily regulatory function, while also recognizing the fluidity between self-regulation and other coping approaches.

We did not intend to organize themes based on the adaptive or maladaptive nature of each coping strategy. Nonetheless, in keeping with prior literature [see (11)], participants described disengagement strategies (e.g., *learned helplessness*) as ultimately enhancing distress and impairment. Some (e.g., *social support, problem-solving*) but not all (e.g., *rumination*) engagement strategies were characterized as protective, contributing toward resilience and post-traumatic growth. Consistently, social support and problem-solving are associated with post-traumatic growth in non-autistic adults (60), whereas brooding rumination may exacerbate traumatic stress symptoms for autistic adults (33). Relatedly, rumination and learned helplessness are known risk factors for depression (61, 62), which itself is a prevalent mental health concern among autistic people (63). Nonetheless, rumination was described by some autistic adults as a way of understanding past events, and by the parents of some children as a way of predicting and preventing future exposure. Consistent with findings in non-autistic samples (64, 65); self-regulatory coping strategies were associated with a mixture of positive and negative outcomes – for example, *engaging in interests* and *self-stimulating/self-soothing behaviors* could bring relief, yet also increase isolation or scrutiny by others. *Self-injurious thoughts and behaviors*, including suicidal ideation, although dangerous and unsustainable in the long-term, were described by participants as bringing momentary relief from their distress – indeed, research has found that both non-suicidal self-injury and suicidal ideation can function to regulate one's emotions, providing temporary reductions in stress and anxiety (66, 67) but also ultimately increasing the risk of a suicide attempt and suicide (68, 69). These nuances highlight the importance of considering coping strategies as having protective *and* detrimental aspects which vary across contexts and timeframes (70, 71), although further investigations are needed to evaluate the generalizability of these qualitative patterns.

We also observed that the utility and accessibility of coping behaviors may depend on personal and contextual factors, including an individual's developmental level, the nature and chronicity of their trauma, and the external resources available to them. *Self-protective behavior, learned helplessness* and *emotional outbursts* were often employed by children and adults in response to chronic stressors which felt beyond the individual's control, such as prolonged bullying or abuse, or a lack of protection from authority figures following trauma disclosure. On the other hand, *problem-solving* and *cognitive restructuring* were often used by older, verbally-able individuals, or those with strong social support networks, including people who recognized and responded to their needs, and who in some cases directly taught them how to use strategies. Overall, our findings suggest that – as with non-autistic people (72) – coping by autistic people takes many forms, may moderate relationships between PTE and outcomes, and is shaped by a confluence of individual and contextual factors. At the same time, existing theories and measures may require adjustment to accommodate additional complexities relevant to autistic people, such as including a broader range of coping behaviors. Systematic investigations of these nuances, particularly the effectiveness of different strategies, will be critical to developing tailored and effective interventions for this group (73). For example, future research could investigate the utility of programs designed to increase access to different forms of social support across multiple settings (e.g., at home, within schools, online, or in the community), as these could provide coping resources to autistic individuals, which may be especially valuable in the context of uncontrollable or chronic trauma. At the same time, programs that teach and scaffold the use of problem-solving and cognitive reframing strategies may prove beneficial in supporting autistic children and adults alike to cope with trauma more effectively, as has been found in other pediatric and neurodevelopmental populations (74–76). Importantly, such programs may be informed by future research seeking to identify the most effective forms of problem-solving or cognitive restructuring, as well as the traumatic contexts in which such strategies are most protective or beneficial.

Though not the primary aim of this investigation, a recurring theme which cut across the narratives of autistic adults and caregivers related to the challenges associated with differentiating coping from autism itself. We termed this theme Diagnostic Overshadowing as it pertained to the ways in which coping strategies may be misunderstood or overlooked due to the presence of an autism diagnosis. Participants described overlap between autistic behaviors and many identified coping strategies, including *social withdrawal, emotional avoidance, emotional outbursts, self-stimulatory/self-soothing behaviors*, and *self-injurious thoughts and behaviors*. *Social withdrawal* was described as a self-preserving response to bullying and marginalization, but at first blush could resemble and therefore be mistaken for differences in social understanding, communication, or motivation. Similarly, *emotional avoidance* could be a manifestation of alexithymia, which commonly affects autistic individuals (77), yet was described by participants as a purposeful attempt to minimize outward emotions, manage stress, and avoid scrutiny. Relatedly, in their conceptualization of masking, Pearson and Rose (78) suggest that for autistic people, a social context of stigma and victimization may underlie the pressure to suppress one's natural cognitive, emotional, and motor responses as a form of self-protection. *Self-stimulatory/self-soothing* behaviors involved modulating one's emotions by engaging in repetitive or sensory-focused activities. Restricted and repetitive activities are designated features of autism (2), yet were described herein as creating moments of relief to manage trauma-related distress. Consistent with these findings, prior research has shown that autistic adolescents and adults describe stimming as an important coping mechanism that helps them self-regulate when distressed (40, 41). Finally, self-injury and emotional outbursts are sometimes conceptualized as challenging behaviors associated with autism, yet were described by participants as outlets through which autistic individuals channeled overwhelming distress or responded to sensory pain. Existing research suggests that overwhelming and painful experiences of sensory stimuli can trigger meltdowns among autistic individuals (79). However, participants in the current study reported a variety of traumas, both sensory and interpersonal, that appeared to underlie emotional outbursts, highlighting the range of potential traumas that may be experienced as physiologically overwhelming and painful.

Diagnostic overshadowing is a barrier to the detection of mental health conditions in autistic individuals (80–82), particularly trauma (43, 83). Consistently, participants in this study illustrated the challenges and implications of traumatic coping being overshadowed for autistic individuals. Parents consistently described the difficulties they and others encountered when attempting to parse their child's traumatic reactions from behaviors commonly associated with autism, an issue also acknowledged by clinical experts (43, 44). Some highlighted the significant ramifications diagnostic overshadowing had in terms of the resources and legal recourse made available to them, while others reflected that not recognizing signs of coping caused themselves, educators, and healthcare workers to miss opportunities to intervene at the peak of their child's distress. These observations are consistent with studies documenting the reduced provision of trauma-focused treatments to autistic versus non-autistic youth, as well as a desire for training in trauma identification and treatment amongst autism-focused providers (29, 84). Similarly, autistic adults reflected on how their attempts to cope could be dismissed as part of their autism diagnosis. Some noted this could increase experiences of stigma and discrimination, which are linked with attempts to mask autistic behaviors and poorer well-being (78). Others highlighted the potential harm of intervention programs that aim to eradicate coping behaviors without considering their protective function.

The overlap between coping and autistic behaviors described here and in prior theoretical work (28, 29) raises important phenomenological questions about their nature, function and intersection. First, it is crucial to note that many of these coping behaviors (e.g., *social withdrawal, emotional outbursts*) are not unique to autism, and have been widely reported in studies of coping in the non-autism population (11). Nonetheless, the overlap of these strategies with autism-related behaviors may minimize this reality, such that the coping function of these behaviors is under-estimated. It may be that autism-related behaviors overlap with, or increase one's propensity toward a particular coping style; for example, emotion regulation difficulties could motivate varied attempts, such as meltdowns or self-injury, to control distress. Another possibility is that behaviors traditionally viewed as traits of autism also serve a self-regulatory function, and reflect autistic people's attempts to cope with stressors (both traumatic and non-traumatic). Recognizing this overlap may provide a more compassionate than pathologising lens through which to understand the experiences and actions of autistic people, with important implications for clinical practice. For example, notable changes in intensity or frequency of restricted and repetitive behaviors, social withdrawal, or emotional outbursts may signal attempts to cope with distress, alerting others to assess for past or ongoing trauma. In addition, intervention programs aiming to manage or reduce behaviors such as stimming may be untenable if they bar an autistic individual from accessing their coping resources. Relatedly, practitioners should consider if social withdrawal reflects a coping strategy that serves a self-preserving function when planning and delivering interventions to enhance social engagement and integration. In such cases, the clinician and client may need to identify alternative coping strategies that can be protective while still promoting social connection. Ultimately, instead of removing behaviors (and potentially valuable coping resources), autism- and trauma-informed care providers should collaboratively identify strategies that are sustainable, adaptive in the long-term and which best fit an individual and their circumstances in a way that respects their autonomy and differences.

Adding further complexity to the picture, many of the reported coping strategies also overlapped with designated symptoms of PTSD (2). For instance, *rumination* may reflect intrusive thoughts or memories of the trauma. *Problem avoidance* could represent a symptom of avoidance. *Self-criticism, social withdrawal*, and *emotional avoidance* could signify negative alterations in cognition and mood. Finally, *emotional outbursts, self-protective behavior*, and *self-injurious thoughts and behaviors* could indicate alterations in arousal and reactivity. To our knowledge, the conceptual and phenomenological distinctions between coping behaviors and PTSD symptoms have not been well-defined, yet research has consistently linked certain strategies (e.g., avoidance-based coping) with the subsequent development and severity of PTSD symptomatology (8, 9). We suggest the possibility that when used protractedly, certain coping strategies can themselves become symptoms of PTSD. Although beyond the scope of the current study, further defining the conceptual and temporal boundaries between coping and psychopathology will be of broader theoretical and clinical value.

### Limitations and Future Directions

Limitations of this study include a reliance on caregiver perspectives to explore the coping of younger children and individuals with communication challenges, which may introduce bias. Further research that systematically gathers and compares multi-informant (e.g., self, parent, teacher) reports of coping behaviors will illuminate converging and diverging perspectives on this phenomenon. Relatedly, we used stratified purposive sampling to select a heterogeneous sample of participants for this study, in order to capture a wide range of perspectives from autistic adults and caregivers. Although this sampling approach facilitated our examination of coping from diverse angles, allowing us to identify common themes across the sample despite its heterogeneity (85), future research investigating coping in more homogeneous samples of autistic people (e.g., among children, or adolescents specifically) may complement our broader observations with more nuanced findings regarding the coping of specific subgroups. On the other hand, limited racial diversity among autistic adults and underrepresentation of autistic girls, non-binary, or transgender individuals in the caregiver sample limited our ability to explore cultural and gender differences in experiences of trauma and coping (86–88). Further, this study focused on coping, rather than the range of traumatic responses possible amongst autistic individuals, which may include trauma resistance (not recognizing an event as traumatic) as well as resilience (risk and protective factors) and symptomatology. It is also important to note that the range of traumatic experiences encountered by autistic individuals varied as widely as the strategies they used to cope. Consistent with prior theoretical work (29) and as has been reported in recent research (44, 46, 52), individuals in this study experienced both PTSD-defined (e.g., physical and sexual abuse, life threatening accident) and broader (e.g., stigma and discrimination, sensory trauma, social confusion) traumas. We suggest the diversity of coping behaviors reported in this study reflects the diversity of traumatic experiences relevant to autistic individuals. Accordingly, future research should examine how coping behaviors may vary in response to traditionally recognized and distinct traumas. Finally, our qualitative observations, while fruitful in generating initial hypotheses and raising questions for further research, require replication and enumeration via quantitative investigation.

## Conclusion

It is increasingly recognized that autistic people are disproportionately vulnerable to trauma. Compounding this vulnerability, a lack of access to autism- and trauma-informed mental healthcare may further contribute to physical and mental health disparities. Yet, our understanding of how autistic people respond to trauma, and whether their attempts to cope moderate post-traumatic outcomes, remains limited. Here, we identified a range of coping strategies used by autistic people, many of which are documented in the non-autism literature, and some of which may be more specific to, or appear to overlap with autism-related behaviors. Our findings highlight important considerations for conceptualizing coping in autism, including factors underlying differences in coping, and how aspects of autism may shape, overlap with, or overshadow coping behavior. Replicating these stakeholder-informed themes in future research may facilitate a more nuanced understanding of how autistic people respond to trauma, informing the development of more accurate measures to assess coping behaviors and their utility, as well as interventions to promote resilience. Ultimately, developing this understanding, supporting individual responses to trauma while also addressing environmental sources of trauma, and learning from the lived experiences of autistic people with trauma histories will be central to providing effective support for this population.

## Data Availability Statement

The original datasets presented in this article are not readily available because they contain potentially identifying or sensitive personal information. Access to the de-identified datasets can be requested on reasonable demand via the senior author at cmkerns@psych.ubc.ca.

## Ethics Statement

The studies involving human participants were reviewed and approved by Drexel University Institutional Review Board. The patients/participants provided their written informed consent to participate in this study.

## Author Contributions

CK led the study conceptualization, study design, and data collection with guidance, and consultation from SL, SB, DR, and CN. AR and HP conducted the data analysis. EN-C, AR, HP, and CK interpreted the results with guidance from TG, and drafted the manuscript. EN-C and CK supervised the research project and writing. All authors contributed to revising the manuscript, including the thematic map, and approved the final version for publication.

## Funding

Funding for this research was provided by the Eunice Kennedy Shriver National Institute for Child Health and Human Development (K23HD07472 to CK), John R. Evans Leaders Funds from the Canadian Foundation for Innovation (#38787 to CK), and the Cordula and Gunther Paetzold Fellowship from the University of British Columbia (to EN-C).

## Conflict of Interest

DR is a co-owner of M-CHAT, LLC. M-CHAT, LLC licenses the use of their intellectual property, the Modified Checklist for Autism in Toddlers (M-CHAT) and M-CHAT Revised, with Follow-Up (M-CHAT-R/F), for use in commercial products and collects royalties. She has a 50% share in the LLC. She also is on the advisory board for Quadrant Biosciences, Inc. The remaining authors declare that the research was conducted in the absence of any commercial or financial relationships that could be construed as a potential conflict of interest.

## Publisher's Note

All claims expressed in this article are solely those of the authors and do not necessarily represent those of their affiliated organizations, or those of the publisher, the editors and the reviewers. Any product that may be evaluated in this article, or claim that may be made by its manufacturer, is not guaranteed or endorsed by the publisher.

## Abbreviations

CSI: Coping Strategies Inventory
DSM-5, Diagnostic and Statistical Manual of Mental Disorders: 5th Edition
PTSD: Post-Traumatic Stress Disorder
PTSS: Post-Traumatic Stress Symptoms
RAADS-14: Ritvo Autism and Asperger Diagnostic Scale-Revised Screen
SAMHSA: Substance Abuse and Mental Health Services Administration
SCQ: Social Communication Questionnaire
TE: Traumatic Experience
THQ: Trauma History Questionnaire.
