# Supplementary material for: A Qualitative Study of Self and Caregiver Perspectives on How Autistic Individuals Cope With Trauma
Source: Front Psychiatry. 2022 Jul 14;13:825008. doi: 10.3389/fpsyt.2022.825008 (PMC9329569; doi:10.3389/fpsyt.2022.825008)
Supplement: Supplementary file 1 [file Table_1.DOCX]

**Brief report: A qualitative study of self and caregiver perspectives on how autistic individuals cope with trauma**

***Supplementary Material***

**Supplementary Table 1. Frequencies of potentially traumatic events (per Trauma History Questionnaire [THQ] and qualitative interviews)^1^**

| *THQ Trauma Items* | **Adult THQ (N=14)** | **Parent THQ (N=15)** |
| --- | --- | --- |
| **Physical abuse** | **11** | **6** |
| Sexual abuse | 5 | 3 |
| **Emotional abuse** | **9** | **8** |
| Serious accident/injury/illness | 5 | 3 |
| **Death of loved one** | **9** | 7 |
| Unexpected separation caregiver | 4 | 6 |
| Traumatic medical treatment | 4 | 4 |
| **Loved one seriously sick/injured** | **9** | 6 |
| Robbery (self/close other) | 4 | 4 |
| Domestic violence | 5 | 5 |
| Community or school violence | 7 | 3 |
| **Bullying** | **14** | **10** |
| Neglect | 4 | 1 |
| Food/Housing Instability | 3 | 4 |
| Exposure to drug/alcohol abuse | 6 | 4 |
| Suicide or attempt in loved one | 5 | 1 |
| Animal attack | 3 | 2 |
| Exposure to disaster | 3 | 4 |
| Incarceration family member | 3 | 0 |
| **“Other trauma”** | **10** | **7** |
| *Note.* Bolding reflects trauma endorsed by ~50% of sample per interviews  ^1^This table has been previously included and reported in Kerns et al. (under review). | | |

**Supplementary Material 2. Qualitative Interview Guide**

*Interview Introduction (Administered by Interviewer)*

As you know, the purpose of this study is to gather information about what kinds of traumatic events individuals with autism experience in childhood and how these experiences influence their lives.

What is trauma? Psychologists define **trauma** as a response to an experience that overwhelms a person’s ability to cope and has a lasting, negative effect on their physical, social or emotional well-being.

We know very little about what experiences result in trauma or what the signs of trauma may be in individuals with autism. Traumatic experiences can be frightening, confusing and upsetting and can lead us to feel sad, mixed-up, scared or angry. They can happen just once or repeatedly over years. Also, many people experience multiple different types of trauma

People respond to traumatic events in very different ways. Also, what is traumatic for one person may not be for another. After a traumatic experience, some people may have difficulties sleeping, feel sad or numb, or want to avoid feelings or reminders of the experience; however, these are only a few of many possible signs of trauma. We are interested in hearing your thoughts about how individuals with autism may experience and express trauma.

*[INSTRUCTIONS: If in-person interview, give participant this written definition of trauma to read. Ask them to explain it back to you as they understand it. Check how this definition compares to what they think trauma is].*

| *Did participant understand trauma definition provided?* Y / N  Describe*:* |
| --- |

| *Describe participant’s personal definition of trauma (if different) here:* |
| --- |

*Traumatic Life Events – Personal Experiences*

*[First ask the below queries to generate a list of potentially traumatic events (PTE). Then ask the participant for more details about each event and its impact on their well-being in PTE boxes.]*

**1. Are there any events or experiences in your childhood that may have been traumatic?**

**2. Are there any other very upsetting events or experiences that seemed to change the way you felt or made you act differently for more than a few weeks?**

**3. Are there any other very upsetting events or experiences like this that happened to you?**

| **Traumatic Experience #1** *(Repeat these questions for all traumatic experiences reported in Q1)*  **A. Description:**  **B. Age of PTE:**  **C. How long did PTE go on:**  **D. Immediate impact (physical, psychological, social, functional):**  **E. Long-term impact (physical, psychological, social, functional):**  **F. Were other people aware of how this trauma affected you? How could they tell?**  **G. Did you try to tell anyone about this? How?**  **H. Were there things you did to help yourself feel better or to get through? Did they help?**  **I. Did anyone else help you to feel better or did you seek treatment? Please describe. Was it helpful? Did you receive any help from school, health providers or other professionals?** |
| --- |

*Traumatic Events – Other Impressions*

**2. Do you think there are there certain types of traumas that people with autism are more likely to experience than people who don’t have autism?**

**3. Do you think there are traumas that people with autism experience that don’t get recognized by other people?**

**4. Do you think people may miss the signs of trauma in people with autism? If so, why?**

**5. Are there things people with autism and those who care about them (family, friends, professionals) could do to reduce the harm caused by traumas or help people recover?**

**6. Did anything change for you in terms of your health care, schooling, living arrangement, work, or involvement other social systems (law enforcement, state services) due to trauma?**

**7. Autism has been chosen as a qualifying condition in PA’s medical marijuana proposition. Do you think medical marijuana may be a helpful treatment for you or others on the spectrum?**

**8. Do you feel your gender/sexuality has played a role in your traumatic experiences?**

**9. Do you feel that understanding your diagnosis of autism may have influence how you experienced or responded to trauma?**

**10. Anything else you think clinicians, researchers, teachers or other health professionals should know/understand?**
